# 

**Published:** 1880-04

**Authors:** 


### CONTENTS.

### COMMUNICATIONS.

Address of Ohio College of Dental Surgery........ ........................ 153
Address of Mississippi Valley Dental Association......................... 165
Dyspepsia and the Teeth........................................................ 170
Discussions at the Thirty-Sixth Annual Meeting of the Mississippi Valley Dental Association............. ................................ 172
Clinical Reports.................................................................... 185

### SELECTIONS.

How to Succeed in our Profession......................... ...................... 186
A Word of Caution in the Administration of Acid Medicines ......... 187
A Case of Hare-Lip.................................................. ......... 188
Women who Smoke............ ................................................ 189
The New Anæsthetic............ ........................................ ............... 190
Wickersheimer's Preservative Fluid............................................ 191
Sarcoma of Jaw-Death.............................................................. 192
How to Test Chloroform.............................................. ....... 193
New York Odontological Society................................................ 194

### EDITORIAL.

Wanted ................................................................................ 195
To the American Dental Association............................................ 195
Tennessee Dental Society......................... ................................ 196
Northern Ohio Dental Association.............................................. 196
Illinois State Dental Society ..... ...... .................................... 196

